# Investigating expectation effects using multiple physiological measures

**DOI:** 10.3389/fpsyg.2015.01553

**Published:** 2015-10-09

**Authors:** Alexander Siller, Wolfgang Ambach, Dieter Vaitl

**Affiliations:** ^1^Clinical and Physiological Psychology, Institute for Frontier Areas of Psychology and Mental HealthFreiburg, Germany; ^2^Bender Institute of Neuroimaging, Justus Liebig, University of GiessenGiessen, Germany

**Keywords:** psychophysiology, expectation, presentiment, consciousness, lie detection

## Abstract

The study aimed at experimentally investigating whether the human body can anticipate future events under improved methodological conditions. Previous studies have reported contradictory results for the phenomenon typically called *presentiment*. If the positive findings are accurate, they call into doubt our views about human perception, and if they are inaccurate, a plausible conventional explanation might be based on the experimental design of the previous studies, in which expectation due to item sequences was misinterpreted as *presentiment*. To address these points, we opted to collect several physiological variables, to test different randomization types and to manipulate subjective significance individually. For the latter, we combined a mock crime scenario, in which participants had to steal specific items, with a concealed information test (CIT), in which the participants had to conceal their knowledge when interrogated about items they had stolen or not stolen. We measured electrodermal activity, respiration, finger pulse, heart rate (HR), and reaction times. The participants (*n* = 154) were assigned randomly to four different groups. Items presented in the CIT were either drawn with replacement (*full*) or without replacement (*pseudo*) and were either presented category-wise (*cat*) or regardless of categories (*nocat*). To understand how these item sequences influence expectation and modulate physiological reactions, we compared the groups with respect to effect sizes for stolen vs. not stolen items. Group *pseudo_cat* yielded the highest effect sizes, and *pseudo_nocat* yielded the lowest. We could not find any evidence of presentiment but did find evidence of physiological correlates of expectation. Due to the design differing fundamentally from previous studies, these findings do not allow for conclusions on the question whether the expectation bias is being confounded with presentiment.

## Exploring anticipatory activity

Mossbridge et al. (2014) defined presentiment as predictive anticipatory activity (PAA), which can be described as an unconscious, non-inferable physiological anticipatory response prior to stimulus presentation. The main assumption of PAA is that the human body can anticipate future events and that we can measure these changes physiologically (Mossbridge et al., 2014). These assumptions fuel the imagination and would, if they are true, call into doubt theories in the fields of psychology and neuroscience that are common in psychophysiological research. In this paper, we will call the phenomenon anticipatory activity (AA), without mentioning the predictive character. We will refer to presponses as unconscious physiological activity preceding an upcoming task or stimulus.

Historically one of the first experimental designs similar to those in recent studies was mentioned by Good (1967) in a letter to the editors of the Journal of Parapsychology. In this letter he mentioned an experiment designed by his brother A. J. Good that was suggested to W. Carington in 1946. In his experiment, a participant should be placed in a dark room in which a light is flashed at random intervals. The collected data should be analyzed to see if an electroencephalography (EEG) reveals a tendency to predict the flashes of light. Levin and Kennedy (1975) conducted a similar physiological experiment, recording EEG data prior to randomly elicited motor responses in a reaction time (RT)-based paradigm in an attempt to predict subjects' responses before the presentation of a green or red light. Hartwell (1978) recorded participants' EEG responses prior to the presentation of pictures of men and women. Current AA experiments often use a design that involves viewing or listening to a series of randomly presented emotional events and neutral events. More recent studies have reported contradictory results and different interpretations of the phenomenon typically called *presentiment*. It has been said that “Anticipatory effects tend to influence baseline values and hence influence the response values” (Bierman and Scholte, 2002, p. 1). Radin (2006) postulates that future, non-inferable experiences unconsciously influence our present physiological state. Explanations for this phenomenon range from physiological to consciousness to quantum biological theories (Mossbridge et al., 2014). Evidence for the existence or non-existence of *presentiment* has been passionately debated for a number of years and has recently been reinvigorated by several independent studies using various psychophysiological measurements, such as skin conductance (Radin, 1997, 2004, 2006; Broughton, 2004; McCraty et al., 2004; May et al., 2005), pupil dilatation (Radin, 2004; Radin and Borges, 2009; Radin et al., 2011; Tressoldi et al., 2011), functional magnetic resonance imaging (Bierman and Scholte, 2002), and electroencephalography (Radin and Lobach, 2007). Bierman (1998) searched for evidence of anticipatory responses in data from previous psychophysiological experiments for various research questions and found differences in skin conductance between dichotomous stimuli preceding their presentation. Radin (2006) reported increased parasympathetic pre-stimulus activity in skin conductance levels before item presentation and between dichotomous stimuli. The latest studies published by Bem (2011) presented a series of experiments specially designed to investigate precognition and explored whether responses could be influenced by future events in a retro causal way. Eight out of nine experiments he conducted yielded statistically significant effects. These experiments were controversial and successfully replicated (Bem et al., 2014) within a meta-analysis of 90 experiments which yielded an overall small effect size of 0.09 (Hedges' g). The results from Bem's experiments on behavioral anticipation effects from 2011 were greatly debated, particularly the statistical issues (Ritchie et al., 2012). Wagenmakers et al. (2011) conducted a Bayesian analysis that revealed that Bem's results were left to chance. On the other hand, (Bem et al., 2011) showed that this analysis underestimated the results and that even using a conservative statistical approach, five out of nine experiments showed a significant effect.

One meta-analysis (behavioral) on forced-choice precognition was published in 1989 and reported significant results and an effect size of *d* = 0.02, averaged from 309 studies (Honorton and Ferrari, 1989). Another AA meta-analysis (psychophysiological) was published in 2012 and reported significant results and an effect size of *d* = 0.21, averaged from 26 published studies (Mossbridge et al., 2012). Schwarzkopf (2014) criticizes previous published *presentiment* studies in five ways. First, he criticized the quality of the studies used for the meta-analysis and the lack of peer reviews of many of them. Second, he states that there should have been more studies not conducted by “psi” researchers. Third, he questions if the ratio of target and control items (mostly around 2:1) affected presponses. Fourth, he questions if presponses are affected by reactions to previous stimuli. Fifth, he states that the effects of expectation must always be tested to check if participants learn regularities in item sequences. Mossbridge et al. (2015) responded to these five critiques, and pointed out that all conference proceedings passed formal peer review, that they couldn't obtain more data from mainstream psychophysiology labs, that because emotional stimuli were less likely to occur, participants would do better predicting calm stimuli and making it more unexpected to find significant presponse differences. Baseline correcting the z-transformed reactions should diminish effects from previous stimuli and if expectation could explain *presentiment* effects there has to be a negative correlation between effect size and number of participants.

## Expectation effects

Previous studies on *presentiment* may have suffered from statistical deficits and the insufficient consideration of expectation and order effects as possible explanations for the phenomenon (Dalkvist et al., 2002; Wackermann, 2002). As mentioned by Mossbridge et al. (2014), taking expectation effects into account is crucial to understand reported outcomes in AA experiments. One potential statistical bias is the so-called “gambler's fallacy,” which is based on a gambler's (false) expectation that the likelihood of something happening less frequently will happen more frequently in the future (or vice versa). According to Dalkvist et al. (2002) referring to *presentiment* experiments, “This theoretically possible behavior could occur if participants believe that the likelihood of the next picture being activating increases as the number of calm pictures shown since the last activating picture increases (that is, ‘the gambler's fallacy’)” (p. 2). In terms of our present experiment, the false or true expectation (depending on the predictability of the item sequences in each group) that the likelihood of a stolen item being presented next increases with the number of previous not stolen items.

In a computational simulation of neutral and emotional events, physiological data showed physiological changes with rising expectations if an imminent emotional trial approached (Dalkvist et al., 2002). Expectation effects in *presentiment* experiments needed to be taken into particular account. For Radin (2004), expectation was a viable explanation for AA if emotional events with a greater number of neutral events preceding them have larger effects than those with fewer neutral events preceding them. This considers only one of the many types of expectation, which is due to order effects. Other plausible expectation effects like temporal expectation are not considered. The temporal predictability of events can directly induce temporal expectation, meaning that a cognitive event to process a stimulus or to press a button after a certain interval can lead to shorter responses for that specific event (Thomaschke and Dreisbach, 2013).

Comparing different types of randomization may result in knowledge about systematic expectation effects caused by item sequences. Physiological reactions due to expectation effects could be falsely interpreted as AA. Emerging from these issues, the objective of this study is to replicate these reported AAs under improved methodological conditions. We opted for the collection of multi-channel physiological variables and an innovative experimental design. Various physiological measures may help to reduce bias, increase statistical power, and bring to light ideas about the nature of physiological changes in AA. The experimental design could help to create a situation closer to real life than other previous experiments. This may also result in new insights about expectation effects and reveal psychophysiological reactions preceding item presentation. The goal is to create a new way to test AA and to comprehend the above-mentioned sources of bias.

## The experiment: forefeeling guilty knowledge

We opted for an experimental design combining the concealed information test (CIT) and a mock crime in which participants are instructed to steal objects from an office. The CIT will be used as a technique to uncover concealed knowledge and to elicit strong physiological reactions related to the presentation of two types of differently significant items. The CIT uncovers an examinee's crime-relevant knowledge based on physiological response differences between crime-relevant and crime-irrelevant items (Lykken, 1959; Ben-Shakhar and Elaad, 2003; Verschuere et al., 2011; Meijer et al., 2014). In interrogations using the CIT, items are generally presented in categories (similar in appearance and/or purpose). Items are randomly presented without replacement (each item is presented only once). This type of presentation probably creates an implicit, predictable, and/or learnable item sequence, inducing physiological correlates of expectation in the participants during the test. Therefore, we decided to break the categories up and created groups with different randomization. We expected that some of the participants would be more vulnerable to expectation effects due to more predictable and/or learnable item sequences. Typically, the CIT is performed in combination with physiological measures, such as skin conductance, electrocardiography, finger pulse, heart rate (HR), and respiration. However, electrooculography, facial electromyography, electroencephalography, and behavioral measures such as RT and body movements are also used. According to Lykken (1959), the CIT relies on psychophysiological correlates produced by the orienting reflex (OR) (Sokolov, 1963), building a connection between behavior and physiology (Barry, 2009). Items in the CIT are particularly significant if recognized by the examinee (crime-relevant) and elicit a stronger OR than items that are not recognized (crime-irrelevant). The OR is known as a cognitive, behavioral, and physiological response to external stimuli that are novel and/or significant (Sokolov, 1963). It allows the subject to get a more detailed understanding of the stimulus and to win time to prepare and respond (Sokolov, 1963). The CIT has been shown to be a valid and trustworthy scientific evaluation technique to spot concealed information and to elicit a strong OR (Ben-Shakhar and Furedy, 1990; Ben-Shakhar and Elaad, 2002, 2003). The combination of a mock crime and a CIT has shown to provide a promising experimental design for unconventional topics (Schönwetter et al., 2011). The combination of mock crime and CIT hasn't been utilized as far as we know to elicit *presentiment* effects; the experimental design has to be considered as an exploratory one and cannot be simply compared with classical *presentiment* experiments.

The mock crime builds a great cover story to introduce participants to the crime-relevant items and to engage them in a presented task. Combined with the CIT, the mock crime allows us to manipulate the personal relevance of crime-relevant vs. crime-irrelevant items experimentally within subjects in a provoking experiment. Items obtain subjective significance by the action (stolen or not stolen) associated with them. The comparison of various types of randomization will reveal expectation and order effects in physiological reactions that could be confounded by AA.

First, we hypothesize that the CIT will show significant differences between stolen vs. not stolen items in all physiological measures. Second, we hypothesize that AA will be found mostly in groups where items were presented with no replacement. Third, by comparing the groups, we will attempt to determine whether the expectation effect is larger when sequences are more predictable and the differences between stolen vs. not stolen items increases as sequence length of not stolen items preceding a stolen item increases.

## Methods

### Participants

A total of 154 participants (110 women and 44 men) divided into four groups participated in this study at the Institute for Frontier Areas of Psychology and Mental Health (IGPP). A power analysis using the G^*^Power 3.1.9.2 computer program (Faul et al., 2007) indicated a 95% chance of detecting a large effect size (defined by Cohen, 1992) between stolen and not stolen items in the response period.

The mean age of our sample was 23.8 (*SD* = 2.9 years), with a range of 19–36 years. Participants were university students from different faculties. All our participants were recruited via student services and bulletins posted in different university faculties and institutes. We excluded psychology and cognitive neuroscience students for the reason of possible bias and knowledge about the experiment. We obtained informed consent from each participant prior to the experiment. Experiments were carried out according to the WMA Declaration of Helsinki—Ethical Principles for Medical Research Involving Human Subjects. We indicated that participation was voluntary and that each participant could quit the experiment at any time without having to give a reason.

### Procedure

Participants had to carry out a mock theft and steal 10 out of 50 randomly designated items from an apparently occupied office. Afterwards, they were interrogated using a computer-based CIT.

We had a total of 50 items divided into 10 categories (office supplies, beverages, kitchenware, wooden fruit, cosmetics, storage boxes, key chains, artificial flowers, clothes, and sweets). The participants were assigned randomly to four different groups. Item sequences in these groups differed as follows: Items in the CIT were presented category-wise with replacement (*full_cat*) or without replacement (*pseudo_cat*) and regardless of categories with replacement (*full_nocat*) or without replacement (*pseudo_nocat*). At the beginning of the experimental session, the participants were preliminarily informed about the topic of the study, the general procedure, and the physiological measurements. After they were informed about the procedure, they were asked to randomly select a paper listing the instructions for the mock crime from a box. Next, they were brought in front of a closed office and then instructed to enter the office, read the selected task, and carry out the instructions. All participants were instructed to steal 10 objects (completely randomized and balanced through the participants). The 10 objects were put in the same places in the office for all participants. The participants were instructed to collect every item in the center of the office, to look closely at every item, and to put them into a suitcase. After they had finished the mock crime, they were instructed to leave the office and go to the laboratory where the second investigator was waiting to interrogate them while taking physiological measurements. After the participants were connected to the physiological recording device, they received instructions for the computer-based CIT. Subjects were informed that they had two different tasks. First, the participants had to answer the CIT questions as fast as possible by pressing “ja” or “nein” (“yes” or “no”) and speaking their answer aloud. Second, they were told to initiate the next trial (next CIT question) by pressing the “weiter” (“next”) key. The participants were informed that someone noticed that they entered the office and saw them walking away with a suitcase. They were instructed to conceal their knowledge about everything they did in the office; in other words, they were instructed to lie, to outwit the test, and to hide their knowledge. A financial reward of 13 Euros plus a 3 Euro bonus was paid to every participant for maintaining a low profile and appearing to be innocent. The experimenter was blind to the conditions and did not know which items were stolen by the participants or to which groups they belonged.

### Physiological recording

Physiological measures were converted from analog to digital at a resolution of 14 bits and logged using the Physiological Data System I 410-BCS manufactured by J&J Engineering (Poulsbo, WA, USA). Physiological data and stimulus onset and offsets were sampled at a ratio of 510 Hz. The behavioral measures were recorded with the same accuracy as the physiological measures and processed for later evaluation of reaction and WTs. Behavioral and physiological data were synchronized with an accuracy of ±2 ms. Skin conductance, respiratory activity, HR, and finger pulse were registered. Skin conductance was measured at a resolution of 0.01 μS. Standard Ag/AgCI electrodes (diameter 0.8 cm), neutral isotonic electrode paste (TD-246, Discount Disposables, Vermont, USA), and a constant voltage of 0.5 V were used. Electrodes were placed at the thenar and hypothenar muscles of the non-dominant hand. Thoracic and abdominal respiration activity was registered using two PS-2 biofeedback respiration belts (KarmaMatters, Berkeley, USA) placed around the upper thorax and the abdomen. HR was measured using Hellige electrodes (diameter 1.3 cm) according to Einthoven II. Finger pulse was recorded on the non-dominant hand with an infrared pulse sensor in a cuff around the end phalanx of the middle finger. The psychophysiological recording was conducted in an acoustically and electrically shielded experimental chamber (Industrial Acoustics Company GmbH, Niederkrüchten, Germany). The light was dimmed and the temperature was maintained by air conditioning at approximately 22.5°C at the beginning of the experiment, with a maximum temperature increase of 1.1°C. The stimuli were presented with a Windows-based computer on a 17-inch monitor with a viewing distance of 70 cm. Subjects sat in an upright position so they could easily reach the keyboard and watch the monitor.

### Data analysis and processing

**Figure 1:**
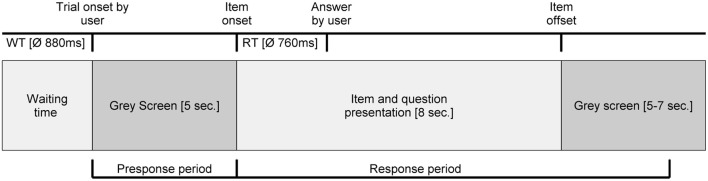
**Experimental procedure of each trial in the computer-based CIT**. RT, Reaction time; WT, Waiting time; ms, milliseconds.

For the data analysis, we defined two different time periods of interest. The first time period (presponse period) was from the time the “weiter” (next) key was hit (trial onset by user) to item onset and was defined to examine presponse differences between reactions to stolen and not stolen items (constant duration of 5 s). The second time period (response period) was from item onset to item offset, suitable for examining response differences between reactions to stolen and not stolen items (duration of 13–15 s) (see Figure 1).

In processing the data, we discarded 603 electrodermal activity trials, 953 respiration trials, 703 HR trials, 1553 finger pulse trials, 585 RT trials, and 727 WT trials out of a total of 7700 trials all physiological and behavioral measure. The authors discarded the data without knowing the group in which the participants were in. The discarded physiological measures were contaminated by different artifacts. Skin conductance data from three subjects were discarded because of electrode flaking and two were discarded because of movement artifacts. Data from skin conductance non-responders were not discarded from the analysis. Respiratory activity data from 12 subjects were discarded because of respiration belt artifacts. HR activity from seven subjects was discarded because of dysrhythmia, extrasystoles, and electrode flaking. Finger pulse data from 25 subjects were discarded because of insufficient signal quality. The behavioral measures were discarded when they exceeded an RT of 3000 ms or fell above 80 ms.

In the presponse period as in the response period, we calculated reaction differences between stolen and not stolen items. Also the same physiological parameters as in the response period were calculated.

Furthermore, we searched for expectation effects in the SCL as an indicator of the arousal of the presponse period. Therefore, we tracked the SCL in the presentation of a series of not stolen (N) items preceding a stolen (S) item. We analyzed item sequences ranging from one to three N items preceding an S item (NS, NNS, and NNNS). The SCL preceding the first N item was subtracted from the SCL preceding the last S item in the sequence. The resulting SCL differences were averaged for each participant and each of the three-item sequences.

For the response period, we analyzed the data according to actual state-of-the-art CIT analysis. Physiological responses in skin conductance (SCR) were defined as an increase in conductance that was initiated within a time period of 1.0–5.0 s after image onset. The amplitude of the response was automatically evaluated as the difference between response onset and the subsequent maximum value in the set time window (Furedy et al., 1991). After low-pass filtering, the total respiration line length (RLL) in the response period was automatically computed over a time interval of 10 s after image onset. The method has been developed by Timm (1982) and was modified by Kircher and Raskin (2003). After notch filtering at 50 Hz, R-wave peaks were automatically detected and visually controlled. The R–R intervals were transformed into HR and real-time scaled (Velden and Wölk, 1987). The HR during the last second before trial onset served as a pre-stimulus baseline. The phasic heart rate (pHR) was calculated by subtracting this value from each second-per-second post-stimulus value. To extract the trial-wise information of the pHR, the mean change in HR within 15 s after trial onset—compared to the pre-stimulus baseline—was calculated (Bradley and Janisse, 1981). From the finger pulse waveform, the finger pulse waveform length (FPWL) within the first 15 s after trial onset was calculated and subjected to further analyses (Elaad and Ben-Shakhar, 2006). The FPWL comprises information about both HR and pulse amplitude and is often interpreted as an indirect measure of arterial blood pressure. The delay between trial onset and the pressing of the key was calculated as RT, the delay between seeing the “next” key and the pressing of the key was calculated as WT. A within-subject standardization of measured values was proposed by Lykken and Venables (1971). Here, according to Ben-Shakhar (1985), Gronau et al. (2005), and Gamer et al. (2006), the physiological and behavioral measures (presponse period and response period) for each subject and data channel were trial-wise z-transformed. These z-transformed values were used for further analysis.

### Statistical analysis

Statistical analyses were performed using SPSS, Version 21.0 (SPSS Inc., Chicago, IL, USA), MATLAB Statistics Toolbox Release 2013a (The MathWorks, Inc., Natick, MA, USA) and G^*^Power 3.1.9.2 (Faul et al., 2007). For the presponse period and the response period, we calculated mean and standard deviations of reactions to stolen and not stolen items. For each physiological and behavioral measure and group, a one sample *t*-test (two-tailed, significance level 0.05) and Cohen's d effect size estimate were calculated. For group comparison, we conducted One-way ANOVAS and *post-hoc* Tukey HSD tests. In order to examine expectation effects in the presponse period, we conducted a one-sample *t*-test for the individual average SCL differences (the SCL preceding the final S item minus the SCL preceding the first N item) and the corresponding effect sizes for each item sequence and group.

## Results

### Overview of physiological measures

**Figure 2:**
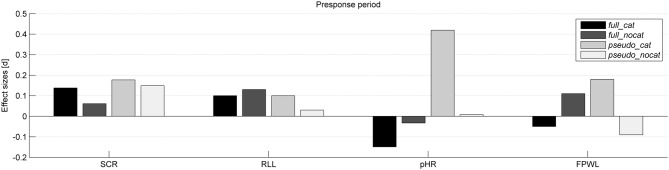
**Effect sizes (Cohen's d) for each physiological measure and group in the presponse period**. SCR, Skin conductance response; RLL, Respiration line length; pHR, Phasic heart rate; FPWL, Finger pulse wave length; group full_cat, Item presentation with replacement in categories; group full_nocat, Item presentation with replacement without categories; group pseudo_cat, Item presentation without replacement in categories; group pseudo_nocat, Item presentation without replacement without categories.

**Figure 3:**
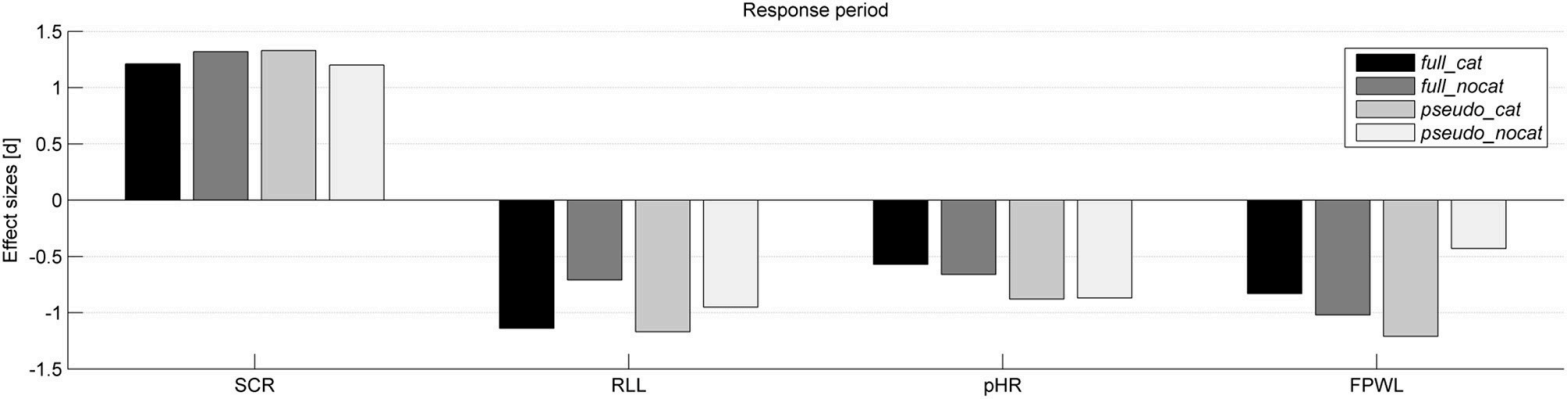
**Effect sizes (Cohen's d) for each physiological measure and group in the response period**. SCR, Skin conductance response; RLL, Respiration line length; HR, Heart rate; FPWL, Finger pulse wave length; group full_cat, Item presentation with replacement in categories; group full_nocat, Item presentation with replacement without categories; group pseudo_cat, Item presentation without replacement with categories; group pseudo_nocat, Item presentation without replacement without categories.

Descriptive statistics based on raw scores are presented before the data standardization and test statistics. Tables 1, 2 summarize the means and standard deviations of the raw scores for each data channel in the presponse period and the response period. Table 3 shows the significance levels and effect sizes for each data channel and group. Table 4 shows the significance levels and effect sizes of SCL preceding stolen items for sequences ranging in length from two to four stimuli. Figures 2, 3 illustrate the effect sizes for each physiological measure in the presponse period and response period, respectively.

**Table 1:** **Means and standard deviations (SD) of raw scores for each data channel in the presponse period**.

**Data channels**	**Stolen**		**Not stolen**	
	**Mean**	**SD**	**Mean**	**SD**
SCR (nS)	149.26	300.58	146.46	315.94
RLL (a.u.)	409.31	606.6	412.92	606.61
pHR (1/min.)	1.46	6	1.43	5.83
FPWL (a.u.)	18247.32	10408.03	18251.5	10598.1
WT (ms)	827.2	412.001	809.84	389.2

*Presponses to stolen and not stolen items. SD, Standard Deviation; SCR, Skin conductance response; RLL, Respiration line length; pHR, Phasic Heart rate; FPWL, Finger pulse wave length; WT, Waiting time; nS, Nanosiemens, a.u., arbitrary units; 1/min., one per minute; ms, milliseconds*.

**Table 2:** **Means and standard deviations (SD) of raw scores for each data channel in the response period**.

**Data channels**	**Stolen**		**Not stolen**	
	**Mean**	**SD**	**Mean**	**SD**
SCR (nS)	545.84	550.33	322.64	433.15
RLL (a.u.)	3115.54	1787.72	3355.53	1903.49
pHR (1/min.)	1	5.24	1.27	5.49
FPWL (a.u.)	14815.23	8621.74	16797.68	9825.38
RT (ms)	1081.81	391.63	1103.97	416.47

*Responses to stolen and not stolen items. SD, Standard Deviation; SCR, Skin conductance response; RLL, Respiration line length; pHR, Phasic heart rate; FPWL, Finger pulse wave length; RT, Reaction time; nS, Nanosiemens; a.u., arbitrary units; 1/min., one per minute; ms, milliseconds*.

**Table 3:** **Significance levels and effect sizes for comparison of responses to stolen vs. not stolen items in the presponse and response period**.

**Group**		**Data channel**	***N***	***df***	***T***	**Sig**.	**Cohen's d**
*Full_cat*—item presentation with replacement in categories	Presponse period	SCR	51	50	0.976	0.334	0.138
		RLL	52	51	0.750	0.457	0.104
		pHR	46	45	−1.017	0.315	−0.15
		FPWL	40	39	−0.598	0.553	−0.049
		WT	51	50	1.035	0.306	0.145
	Response period	SCR	51	50	7.972	0.000^***^	1.21
		RLL	49	48	−7.971	0.000^***^	−1.14
		pHR	50	49	−4.957	0.000^***^	−0.71
		FPWL	46	45	−5.599	0.000^***^	−0.83
		RT	51	50	−1.587	0.119	−0.22
*Full_nocat*—item presentation with replacement without categories	Presponse period	SCR	50	49	0.422	0.675	0.061
		RLL	51	50	0.936	0.354	0.131
		pHR	48	47	−0.229	0.820	−0.033
		FPWL	43	42	0.694	0.492	0.106
		WT	47	46	0.865	0.392	0.130
	Response period	SCR	47	46	9.078	0.000^***^	1.32
		RLL	44	43	−4.687	0.000^***^	−0.71
		pHR	47	46	−5.535	0.000^***^	−0.8
		FPWL	41	40	−6.526	0.000^***^	−1.02
		RT	47	46	1.484	0.145	0.22
*Pseudo_cat*—item presentation without replacement in categories	Presponse period	SCR	24	23	0.853	0.403	0.18
		RLL	25	24	0.505	0.618	0.100
		pHR	24	23	2.013	0.056	0.411
		FPWL	24	23	0.885	0.386	0.181
		WT	24	23	−0.720	0.479	−0.147
	Response period	SCR	23	22	6.370	0.000^***^	1.33
		RLL	23	22	−5.623	0.000^***^	−1.17
		pHR	23	22	−3.520	0.002^**^	−0.73
		FPWL	21	20	−6.302	0.000^***^	−1.21
		RT	24	23	−6.500	0.000^***^	−1.33
*Pseudo_nocat*—item presentation without replacement without categories	Presponse period	SCR	24	23	−0.719	0.480	0.15
		RLL	26	25	0.154	0.879	0.030
		pHR	24	23	0.004	0.997	0.0007
		FPWL	18	17	−0.395	0.697	−0.093
		WT	23	22	0.636	0.531	0.133
	Response period	SCR	23	22	5.771	0.000^***^	1.2
		RLL	21	20	−4.343	0.000^***^	−0.95
		pHR	22	21	−2.293	0.032^*^	−0.5
		FPWL	17	16	−1.782	0.094	−0.43
		RT	23	22	−0.767	0.451	−0.16

*SCR, Skin conductance response; RLL, Respiration line length; pHR, Phasic heart rate; FPWL, Finger pulse wave length; RT, Reaction time; WT, Waiting time*.

* p < 0.05

** p < 0.01

*** *p < 0.001*.

**Table 4:** **Significance levels and effect sizes of SCL differences occurring in the three item sequences, by groups**.

**Group**	**Sequence**	***N***	***df***	***T***	**Sig**.	**Cohen's d**
*Full_cat*—item presentation with replacement in categories	NS	52	51	−2.544	0.014^*^	−0.712
	NNS	52	51	−0.526	0.601	−0.147
	NNNS	52	51	−0.422	0.675	−0.118
*Full_nocat*—item presentation with replacement without categories	NS	51	52	−1.280	0.207	−0.362
	NNS	51	52	−0.241	0.811	−0.068
	NNNS	51	52	−0.105	0.917	−0.029
*Pseudo_cat*—item presentation without replacement in categories	NS	25	24	−2.196	0.038^*^	−0.896
	NNS	25	24	−2.713	0.012^*^	−1.107
	NNNS	25	24	−3.463	0.002^**^	−1.143
*Pseudo_nocat*—item presentation without replacement without categories	NS	26	25	−0.865	0.395	−0.346
	NNS	26	25	−0.559	0.581	−0.223
	NNNS	26	25	−0.247	0.807	−0.098

N, Not stolen; S, Stolen;

* p < 0.05

** *p < 0.01*.

### Skin conductance

**Figure 4:**
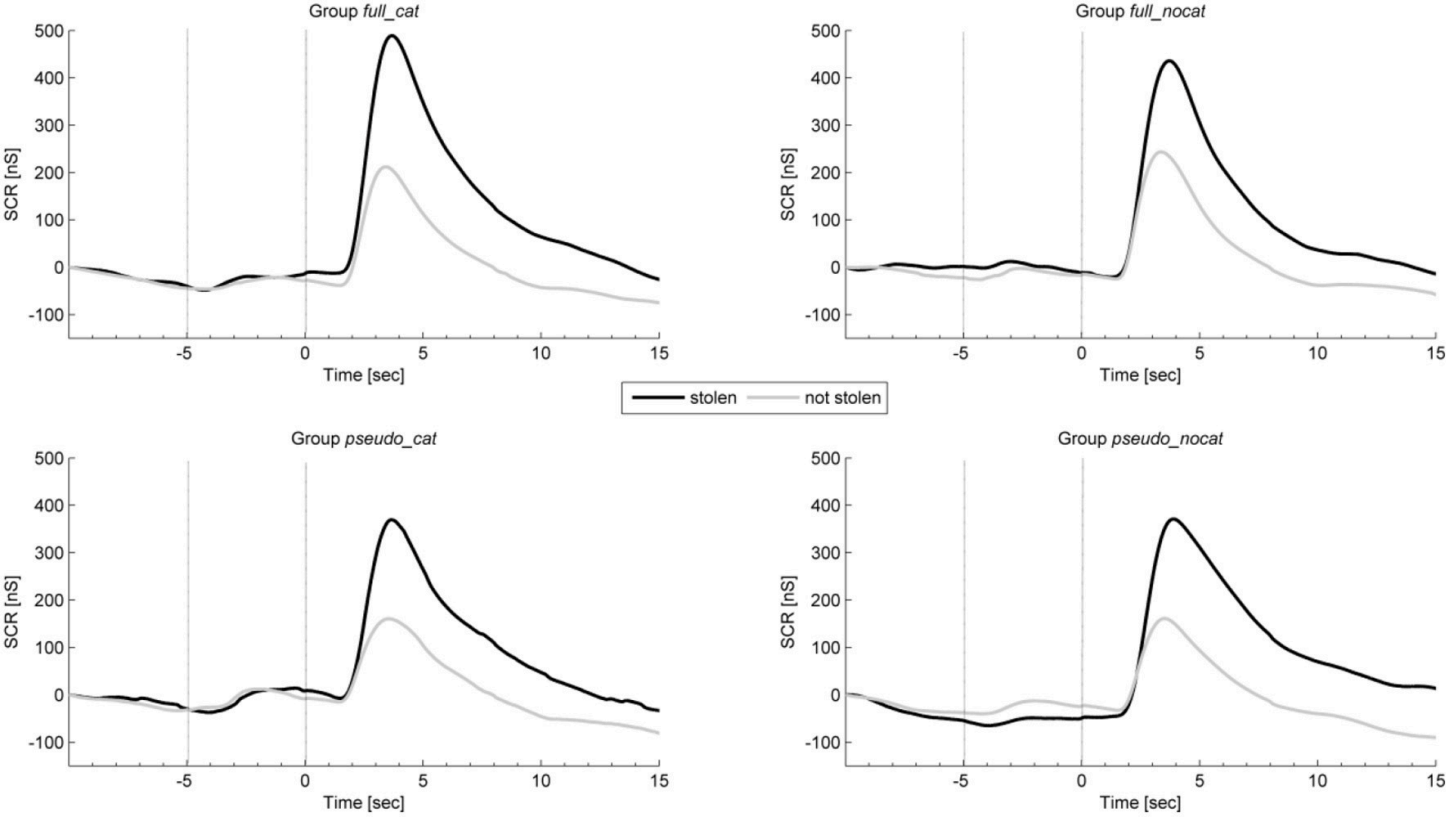
**Mean physiological presponses (time window: −5 to 0 s) and responses (time window: 0 to 5 s) for skin conductance to stolen and not stolen items in each group**. Vertical lines delimit trial onset (sec −5) and image onset (sec 0). SCR, Skin conductance response; group full_cat, Item presentation with replacement in categories; group full_nocat, Item presentation with replacement without categories; group pseudo_cat, Item presentation without replacement in categories; group pseudo_nocat, Item presentation without replacement without categories; nS, Nanosiemens; sec, seconds.

Figure 4 shows the average intra-trial course of skin conductance, depicting grand means for trials with stolen and not stolen items. Grand means show after image onset a strong response amplitude to stolen items exceeding those to not stolen items in each group. Presponse amplitudes between trial and image onset show differences between stolen and not stolen items.

In the presponse period, SCR did not differ significantly between reactions to the presentation of stolen and not stolen items (*M* = 149.26, *SD* = 300.58; *M* = 146.46, *SD* = 315.94); *t*_(142)_ = 0.916, *p* > 0.05, *d* = 0.08. SCR had the highest effect size in group *pseudo_cat* (*d* = 0.18) and lowest effect size in group *full_nocat* (*d* = 0.06).

In the response period, the SCR amplitudes were larger for stolen items than for not stolen items (*M* = 545.84, *SD* = 550.33; *M* = 322.64, *SD* = 433.15); *t*_(142)_ = 14.35, *p* < 0.001, *d* = 1.2. SCR had the highest effect size among all physiological and behavioral measures and showed the highest and lowest effect size in groups *pseudo_cat* (*d* = 1.33) and *pseudo_nocat* (*d* = 1.2), respectively.

In the response period, a One-way ANOVA was conducted to compare the groups. There were no significant difference in SCR between the four groups; *F*_(3, 142)_ = 0.580, *p* > 0.05.

### Respiration

**Figure 5:**
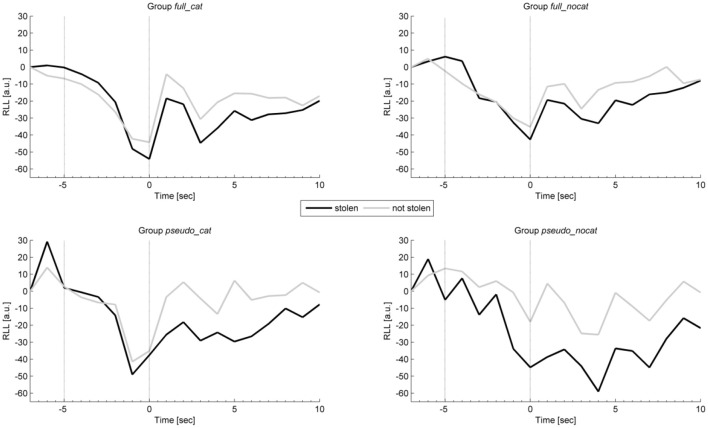
**Mean physiological presponses (time window: −5 to 0 s) and responses (time window: 0 to 5 s) for respiration line length to stolen and not stolen items in each group**. Vertical lines delimit trial onset (sec −5) and image onset (sec 0). RLL, Respiration line length; group full_cat, Item presentation with replacement in categories; group full_nocat, Item presentation with replacement without categories; group pseudo_cat, Item presentation without replacement in categories; group pseudo_nocat, Item presentation without replacement without categories; a.u., arbitrary units, sec, seconds.

Figure 5 shows the average intra-trial course of respiration, depicting grand means for trials with stolen items and trials with not stolen items. Grand means show after image onset a strong response amplitude to not stolen items exceeding those to stolen in each group. Presponse amplitudes between trial and image onset show slight differences; the highest observable difference can be seen in group *pseudo_nocat*.

In the presponse period, the RLL did not differ significantly between reactions to the presentation of stolen and not stolen items (*M* = 409.31, *SD* = 606.6; *M* = 412.92, *SD* = 793.49), *t*_(137)_ = 1.217, *p* > 0.05, *d* = 0.01. The RLL showed the highest and lowest effect size in groups *full_nocat* (*d* = 0.131) and *full_cat* (*d* = 0.104), respectively.

In the response period, the mean RLL response levels were lower for stolen items than for not stolen items (*M* = 3115.54, *SD* = 1787.72; *M* = 3355.53, *SD* = 1903.49), *t*_(137)_ = −11.14, *p* < 0.001, *d* = 1.95. The RLL had the second highest effect size among all physiological and behavioral measures and showed the highest and lowest effect size in groups *pseudo_cat* (*d* = 1.17) and *full_nocat* (*d* = 0.71), respectively.

In the response period, a One-way ANOVA was conducted to compare the groups. There were no significant differences in RLL between the four groups; *F*_(3, 137)_ = 0.075, *p* > 0.05.

### Heart rate

**Figure 6:**
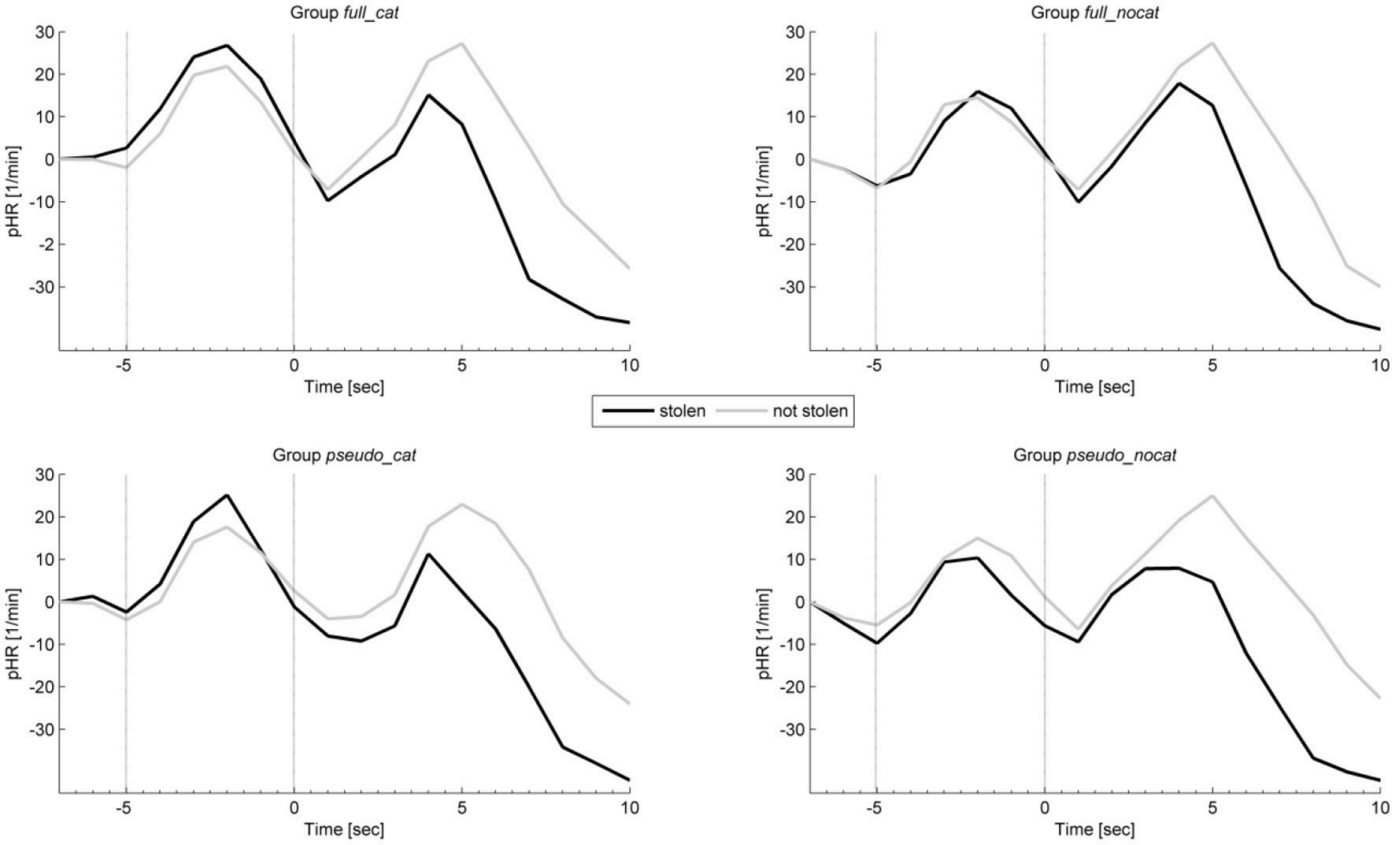
**Mean physiological presponses (time window: −5 to 0 s) and responses (time window: 0 to 5 s) phasic heart rate to stolen and not stolen items in each group**. Vertical lines delimit trial onset (sec −5) and image onset (sec 0). pHR, Phasic heart rate; group full_cat, Item presentation with replacement in categories; group full_nocat, Item presentation with replacement without categories; group pseudo_cat, Item presentation without replacement in categories; group pseudo_nocat, Item presentation without replacement without categories, 1/min, one per minute; sec, seconds.

Figure 6 shows the average intra-trial course of the HR, depicting grand means for trials with stolen items and trials with not stolen items. Grand means show after image onset a strong response amplitude to not stolen items exceeding those to stolen in each group. Presponse amplitudes between trial and image onset show slight differences; the highest observable difference can be seen in group *pseudo_nocat*.

In the presponse period, the pHR did not differ significantly between reactions to the presentation of stolen and not stolen items (*M* = 1.46, *SD* = 6; *M* = 1.43, *SD* = 5.84); *t*_(142)_ = −0.094, *p* > 0.05, *d* = 0.007. The pHR had the highest effect size in group *pseudo_cat* (*d* = 0.41) and the lowest effect size in group *pseudo_nocat* (*d* = 0.007).

In the response period, the pHR response amplitudes were lower for stolen items than for not stolen items (*M* = −1.35, *SD* = 5.95; *M* = 0.26, *SD* = 5.97), *t*_(142)_ = −8.482, *p* < 0.001, *d* = 0.71. Effect sizes in the pHR showed the lowest effect sizes in groups *full_cat* and *pseudo_cat* among all physiological measures and showed the highest and lowest effect sizes in groups *pseudo_cat* (*d* = 0.73) and *full_nocat* (*d* = 0.8), respectively.

In the response period, a One-way ANOVA was conducted to compare the groups. There was no significant difference in pHR between the four groups; *F*_(3, 142)_ = 0.486, *p* > 0.05.

### Finger pulse

**Figure 7:**
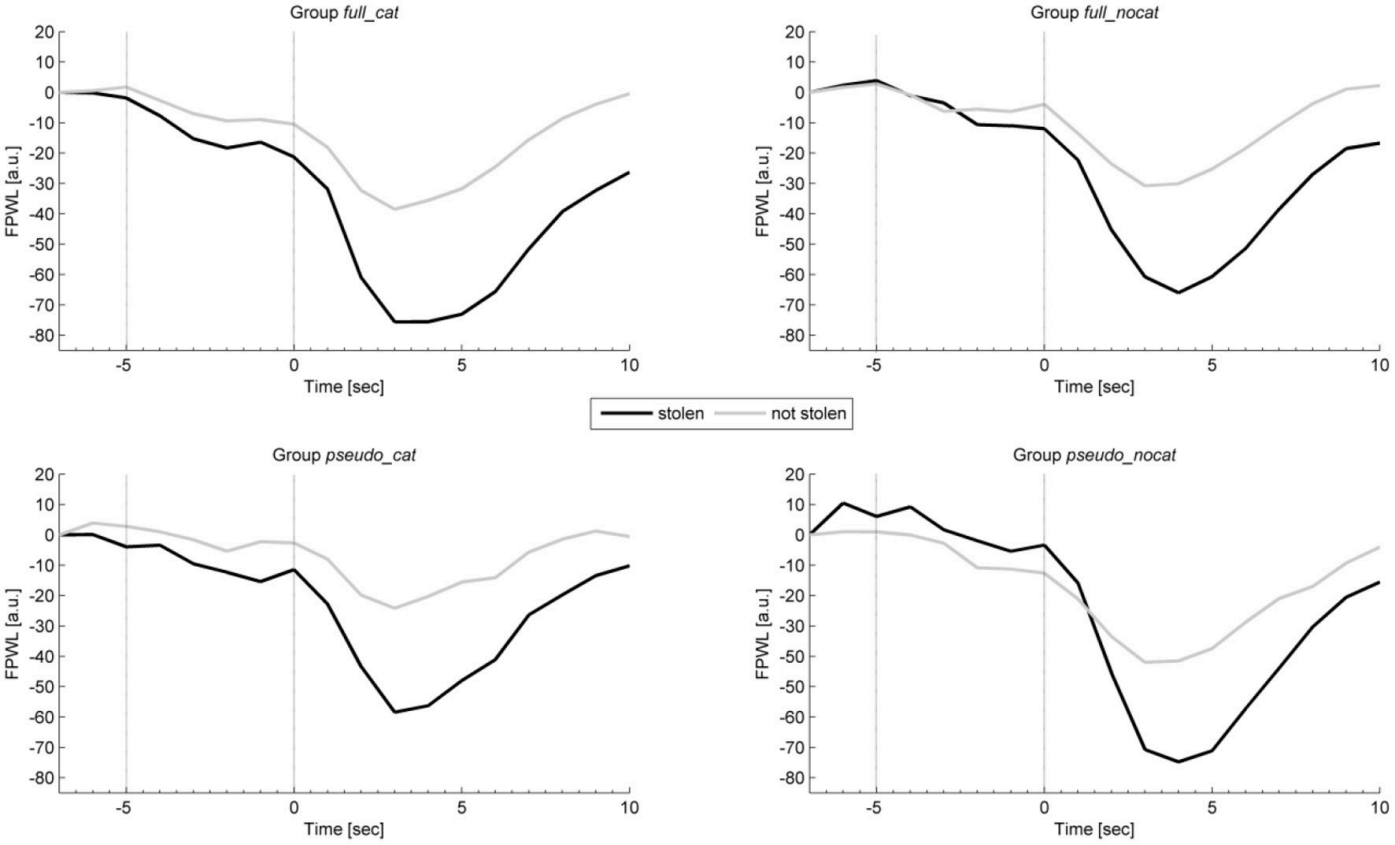
**Mean physiological presponses (time window: −5 to 0 s) and responses (time window: 0 to 5 s) for finger pulse waveform length to stolen and not stolen items in each group**. Vertical lines delimit trial onset (sec −5) and image onset (sec 0). FPWL, Finger pulse wave length; group full_cat, Item presentation with replacement in categories; group full_nocat, Item presentation with replacement without categories; group pseudo_cat, Item presentation without replacement in categories; group pseudo_nocat, Item presentation without replacement without categories; a.u., arbitrary units; sec, seconds.

Figure 7 shows the average intra-trial course of the finger pulse, depicting grand means for trials with stolen items and trials with not stolen items. Grand means show after image onset a strong response amplitude to not stolen items exceeding those to stolen in each group. Presponse amplitudes between trial and image onset show slight differences.

In the presponse period, FPWL response levels did not differ significantly between reactions to the presentation of stolen and not stolen items (*M* = 18247, *SD* = 10408; *M* = 18251, *SD* = 10598), *t*_(142)_ = 0.862, *p* > 0.05, *d* = 0.07. FPWL showed the highest effect size in group *pseudo_cat* (*d* = 0.18) among all physiological and behavioral measures and showed the highest and lowest effect size in groups *pseudo_cat* (*d* = 0.18) and *full_nocat* (*d* = 0.05), respectively.

In the response period, FPWL response amplitudes were lower for stolen items than for not stolen items (*M* = 14815, *SD* = 8621; *M* = 16797, *SD* = 9825), *t*_(125)_ = −9.79, *p* < 0.001, *d* = 0.88. FPWL showed the lowest effect sizes among all physiological measures and showed the highest and lowest effect sizes in groups *pseudo_cat* (*d* = 1.21) and *pseudo_nocat* (*d* = 0.43), respectively.

In the response period, a One-way ANOVA was conducted to compare the groups. There was no significant difference in FPWL between the four groups; *F*_(3, 125)_ = 1.109, *p* > 0.05.

### Reaction time

The mean RT time to CIT items was 1010 ms (max. RT = 2990 ms and min. RT = 243 ms), with a standard deviation of 410 ms. Participants' mean reactions were shorter for stolen items than for not stolen items over all groups. A one sample *t*-test was conducted to compare RTs for stolen items and not stolen items. There was a significant difference in the scores for stolen (*M* = 1082, *SD* = 392) and not stolen (*M* = 1104, *SD* = 416) items; *t*_(144)_ = −2.033, *p* < 0.05. Participants' RTs were shorter when information was concealed.

A One-way ANOVA was conducted to compare the groups. There was a significant difference in RT between the four groups; *F*_(3, 144)_ = 4.705, *p* < 0.05.

*Post-hoc* analysis using the Tukey HSD test indicated that the mean score in the *pseudo_cat* group were significantly different than that in the group *full_nocat*.

### Waiting time

The waiting time (WT) is the time a participant waited until he initiated the next trial. The mean WT before the participant initiating the next trial was 813 ms, with a standard deviation of 394 ms. A one sample *t*-test was conducted to compare the WTs for stolen and not stolen items before item presentation. There were no significant differences in WTs for stolen items (*M* = 827.20, *SD* = 412.001) and WTs for not stolen items (*M* = 809.84, *SD* = 389.198); *t*_(144)_ = 1.414, *p* > 0.05. Participants' WT did not differ significantly for stolen and not stolen items.

A two-tailed One-way ANOVA was conducted to compare the groups. There was a no significant difference in WT between the four groups; *F*_(3, 144)_ = 0.505, *p* > 0.05.

### Item sequences in the presponse period

Table 4 summarizes the results of the one-sample *t*-tests and effect sizes of the SCL differences occurring in the presentation of each of the three-item sequences separately for each group. There was a significant difference in the SCL in the *full_cat* group for the NS item sequence and in the *pseudo_cat* group for the NS, NNS, and NNNS item sequences. The effect sizes in the *pseudo_cat* group tended to decrease with the increasing number of N items preceding the S item, but this did not occur in the other groups.

## Discussion

The aim of the present study was to investigate whether the human body can anticipate future events under varied randomizations. To achieve this goal, we modified the CIT and varied item categorization and randomization type.

We investigated four different groups: first, the *full_nocat* group (item presentation with replacement without categories), consisting of conventionally non-predictable item sequences to test AA and allow for comparison with previous studies; second, the *full_cat* group (item presentation with replacement and categories) to test the influence of categories on expectation when items are presented with preplacement; third, the *pseudo_cat* group (item presentation without replacement and categories), consisting of highly predictable item sequences to test expectation effects; and fourth, the *pseudo_nocat* (item presentation without replacement and categories) to test the influence of categories on expectation when items are presented without replacement.

These four groups were tested to help us answer the following questions: How do categories and item randomization affect physiological reactions? Does AA occur in the new CIT paradigm? Can expectation effects help to explain the differences in physiological reactions between the groups? Can we find AA and/or expectation effects even in the groups without any predictable sequence?

### Anticipatory activity in the concealed information test under varied conditions

In the presponse period, we could not find significant reaction differences between *stolen* and *not stolen* in any of the four groups. Our *full_nocat* group, consisting of non-predictable sequences, was the most comparable to previous *presentiment* studies and showed the closest effect sizes in respiration (*d* = 0.131) and finger pulse (*d* = 0.106) to those in Mossbridge et al. (2012) (*d* = 0.21); heart rate (*d* = 0.061) and skin conductance (*d* = −0.033) were clearly different. One possible explanation for not reaching the level of statistical significance could be that our experiment was underpowered. However, we should exercise caution because the *p*-value does not provide information about the size or strength of the effect. Coulson (2010) could show that statistics considered as significant easily influenced the reader to believe the effect truly exists (which always includes the possibility of a false-positive result). Therefore, as Cumming (2010) suggested, the reader should attend to confidence intervals and effect sizes as warrants of replication. Unlike the group with the most unpredictable item sequences, the *pseudo_cat* group with highly predictable item sequences, showed the highest effect sizes in HR, skin conductance, and finger pulse over all groups. Even if these effects are not significant, we expected to find the highest effect sizes in the *pseudo_cat* group in which the participants received the most cues regarding which item was coming next. Reaction differences seem to increase as item sequences become more predictable. Another interesting finding in support of this assumption is that in the *pseudo_cat*, HR exceeded Cohen's d convention for a small effect size (*d* = 0.41). One possible explanation for this effect can be found in Jennings and Hall (1980), who defined HR responses “as a function of changes in the accessibility of processing capacity” (p. 43). Accordingly, it is believed that HR acceleration reflects ongoing cognitive processing. Some studies have shown that HR constitutes a different working process that is different (Barry and Maltzman, 1985) but parallel working (Gamer et al., 2008) to the orienting reflex. Thus, HR is being exposed as a possible indicator of ongoing cognitive processing. This reinforces our assumption that the more cues the participant gets about the upcoming item, the more expectation is built.

Presenting the items with or without categories seemed to make a difference. The *full_cat* group showed considerably higher effect sizes in skin conductance (*d* = 0.138) and heart rate (*d* = −0.15) than *the full_nocat* group without categories (*d* = 0.061) and (*d* = −0.0339), respectively. Even if these effect sizes are lower than Cohen's d convention for a small effect size, they reflect the effect we found in the response period and could be indicators of expectation. One reason for these effect sizes could be that the differences between the cues given by categories vs. no categories were too subtle. Another possibility is that inter-individual physiological response differences in the manifestation of expectation make it difficult to follow up underlying response patterns and to identify them as correlates of expectation.

Perhaps as you would have thought, we could not find any significant effects for AA in the group with less predictable item sequences; however, we were unable to find AA in any of the other three groups either.

It is interesting that even the *pseudo_cat* group didn't show significant results with the most predictable item sequences. It is important to mention that while our analysis does not necessarily reflect AA, it may be that this phenomenon is present in the data and a different analysis would bring it to light.

### Item sequences in the presponse period

As previously explained, we could not find any evidence of AA; however, even though we could not find any correlates for AA, we wanted to understand the physiological reaction differences between groups (even when those differences were not significant) and reveal underlying expectation effects as a possible explanation for these differences. When we analyzed the item sequences, we tried to show the “gambler's fallacy” in terms of our present experiment: The (false) expectation that the likelihood of a *stolen* item being presented next increases with the number of previously presented *not stolen* items, and this is transferable to physiological reactions and can be manipulated through different randomizations.

This analysis could give us a deeper understanding of how sequences influence physiological reactions. Interestingly, the effect sizes in the group with the most predictable item sequences (*pseudo_cat*) tended to increase significantly as number of *not stolen* items preceding the *stolen* items increased. As an indicator of expectation, the skin conductance level decreased as the number of *not stolen* items increased when the participant had more cues about the upcoming item. This finding encourages us to believe that, first, the more predictable an item sequence is, the more expectation is built; second, the longer this sequence is, the higher the skin conductance level differences will become. Furthermore, the findings indicate that categorization and randomization and the resulting item sequences influence psychophysiological reactions.

Interestingly, this effect is shown the other way around in the other three groups (*full_nocat, full_cat*, and *pseudo_nocat*), where the effect sizes tended to decrease as the number of *not stolen* items preceding the *stolen* item increased. In the *full_cat* group, the shortest item sequence (a *not stolen* item followed by a *stolen* item) showed a significant medium effect sizes (*d* = −0.712). Interestingly, when the categories were resolved (*full_nocat*), this effect dropped to a small effect size (*d* = −0.362) and was no longer significant. This shows that even slightly better predictability or perceived control regarding which item is going to be presented next can influence psychophysiological reactions. As the number of items increased, the effect sizes tended to decrease less in the *full_cat* group than in the *full_nocat* group.

Independent of these findings, the “gambler's fallacy” cannot be fully excluded in the *full_nocat* group, and even if the participant had no cues about the upcoming item, they still could build expectation.

### Differential concealed information test responding

As we expected, the physiological reaction differences in the response period were significant in each data channel and group. Reaction time was only significant in the *pseudo_cat* group in which the answers to *stolen* items were faster than to *not stolen* items. It is likely that the differences in the reaction time were only significant in this group due to the easier predictability of the upcoming item.

The largest effect sizes in the response period for all groups were as follows: skin conductance, respiration, finger pulse, and HR. These results are comparable with previous similar experiments. After comparing the response period in the groups, the largest effect sizes were found in the groups with categories, and the largest effect sizes overall were found in the *pseudo_cat* group. The combination of categories and item presentation without replacement seems to produce the highest effect sizes in the response period. We can assume that item presentation without replacement and categories boosts the psychophysiological reaction differences between *stolen* and *not stolen* items in the CIT.

Item presentation without replacement seems to be more effective for eliciting larger reaction differences between the presentation of *stolen* and *not stolen* items than item presentation with replacement.

In terms of the CIT, we could conclude that more cues about the upcoming item could lead to larger reaction differences between *stolen* and *not stolen* items. Furthermore, our results show that categorization, randomization and the resulting item sequences influence psychophysiological reactions. As a possible explanation, the increased sense of control produced by increased predictability, and the resulting sense of expectation, seems to potentiate reaction differences in the CIT. In other words, as we were able to show in the sequence analysis and again here, the more cues the participant has about the upcoming item, the larger the expectation and physiological reactions become.

## Conclusions

If the human body could anticipate future events, it would change our view about consciousness and behavior. Studies have yielded different interpretations of their results and have opened a debate about this phenomenon. Many of these experiments focus on comparing physiological reactions to a series of randomly presented emotional and neutral items, where expectation was taken into account as a source of bias. Still, underlying inter-individual differences in physiological response patterns, according to different patterns of expectation, cannot be fully excluded. Given the extensive range of implications of the results mentioned in the meta-analyses and in other publications about *presentiment*, discussion about the significance of the findings should be encouraged.

In our experiment, we could not find any evidence for the phenomenon of *presentiment*. However, as the CIT hasn't shown *presentiment* effects in previous literature, it is possible that the methodology wasn't adequate for eliciting *presentiment*. To understand the reaction differences between the groups, we analyzed the item sequences in the presponse period, which showed that the influence of item sequences on the physiology lies deep in the data and could easily have been overlooked in previous similar experiments.

Still, it was not possible to conclude that expectation effects are being confounded with *presentiment*; even when item sequences were unpredictable, they could still be influenced by ongoing cognitive processing that is not based on objective probabilities. The gambler's fallacy cannot be reliably excluded and should be further experimentally investigated.

Nonetheless, the experiment suggests that expectation can affect physiological reactions: predictable item sequences showed larger reaction differences than groups with less predictable item sequences. The expectation effect increases as the sequence length of *not stolen* items preceding *stolen* items increases.

Non-predictable or less predictable item sequences showed smaller reaction differences.

It is reasonable to assume that when participants have more cues to help them guess which item is next, more expectation is built; the fewer cues they have, the less expectation is built.

Even if our experiment shows that participants' reactions to stimuli change due to item sequences, a more promising approach to understanding expectation effects might be to design an experiment that specifically tests expectation. In such an experiment, participants would be exposed to different stimulus sequences different numbers of cues about the upcoming item sequences. This could help to understand expectation effects in a more fundamental way. It could also contribute to an understanding of the phenomenon of *presentiment*, which could be explainable as a different type of expectation or as an unconventional explainable phenomenon.

Apart from the issue of *presentiment*, the results of this experiment reflect the importance of considering the effects of item sequence and randomization in classic stimulus-reaction experiments as a possible source of bias, and they provide new theoretical and practical insights for psychophysiological experiments and the CIT.

### Conflict of interest statement

The authors declare that the research was conducted in the absence of any commercial or financial relationships that could be construed as a potential conflict of interest.

## References

[B1] Barry R. J. (2009). Habituation of the orienting reflex and the development of preliminary process theory. Neurobiol. Learn. Mem. 92, 235–242. 10.1016/j.nlm.2008.07.007 18675927

[B2] Barry R. Maltzman I. (1985). Heart rate deceleration is not an orienting reflex; Heart rate acceleration is not a defensive reflex. Pavlov. J. Biol. Sci. 20, 15–28. 3982868 10.1007/BF03003235

[B3] Bem D. J. (2011). Feeling the future: experimental evidence for anomalous retroactive influences on cognition and affect. J. Pers. Soc. Psychol. 100, 407–425. 10.1037/a0021524 21280961

[B4] Bem D. J. Utts J. Johnson W. O. (2011). Must psychologists change the way they analyze their data? J. Pers. Soc. Psychol. 101, 716–719. 10.1037/a0024777 21928916

[B5] Bem D. Tressoldi P. Rabeyron T. Duggan M. (2014). Feeling the Future: A Meta-analysis of 90 Experiments on the Anomalous Anticipation of Random Future Events. Daryl J. Bem Cornell University. Available online at: http://dbem.us/FF%20Meta-analysis%206.2.pdf 10.12688/f1000research.7177.1 PMC4706048 26834996

[B6] Ben-Shakhar G. (1985). Standardization within individuals: a simple method to neutralize individual differences in skin conductance. Psychophysiology 22, 292–299. 10.1111/j.1469-8986.1985.tb01603.x 4011799

[B7] Ben-Shakhar G. Elaad E. (2002). The Guilty Knowledge Test (GKT) as an application of psychophysiology: future prospects and obstacles, in Handbook of Polygraph Testing, ed Kleiner M. (San Diego, CA: Academic Press), 87–102.

[B8] Ben-Shakhar G. Elaad E. (2003). The validity of psychophysiological detection of information with the guilty knowledge test: a meta-analytic review. J. Appl. Psychol. 88, 131–151. 10.1037/0021-9010.88.1.131 12675401

[B9] Ben-Shakhar G. Furedy J. J. (1990). Theories and Applications in the Detection of Deception: A Psychophysiological and International Perspective. New York, NY: Springer-Verlag.

[B10] Bierman D. (1998). Do psi phenomena suggest radical dualism?, in Toward a Science of Consciousness II, eds Hameroff S. R. Kaszniak A. W. Scott A. C. (Cambridge, MA: MIT Press), 709–714.

[B11] Bierman D. J. Scholte H. S. (2002). Anomalous anticipatory brain activation preceding exposure of emotional and neutral pictures, in Toward a Science of Consciousness, Tucson IV.

[B12] Bradley M. T. Janisse M. P. (1981). Accuracy demonstrations, threat, and the detection of deception: cardiovascular, electrodermal, and pupillary measures. Psychophysiology 18, 307–315. 10.1111/j.1469-8986.1981.tb03040.x 7291448

[B13] Broughton R. (2004). Exploring the reliability of the “presentiment” effect,” in Proceedings of the 47th Convention of the Parapsychological Association. Ultrecht, Holland Available online at: from http://archived.parapsych.org/papers/02.pdf

[B15] Cohen J. (1992). A power primer. Psychol. Bull. 112, 155–159. 10.1037/0033-2909.112.1.155 19565683

[B14] Coulson M. (2010). Confidence intervals permit, but don't guarantee, better inference than statistical significance testing. Front. Psychol. 1:26. 10.3389/fpsyg.2010.00026 21607077 PMC3095378

[B16] Cumming G. (2010). *p*-values versus confidence intervals as warrants for conclusions that results will replicate, in Methodologies for conducting research on giftedness, 1st Edn., eds Thompson B. Subotnik R. (Washington, DC: American Psychological Association), 53–69.

[B17] Dalkvist J. Westerlund J. Bierman D. J. (2002). A computational expectation bias as revealed by simulations of presentiment experiments, in Proceedings of the 45th Annual Convention of the Parapsychological Association, 62–79.

[B18] Elaad E. Ben-Shakhar G. (2006). Finger pulse waveform length in the detection of concealed information. Int. J. Psychophysiol. 61, 226–234. 10.1016/j.ijpsycho.2005.10.005 16712993

[B19] Faul F. Erdfelder E. Lang A.-G. Buchner A. (2007). G^*^ Power 3: a flexible statistical power analysis program for the social, behavioral, and biomedical sciences. Behav. Res. Methods, 39, 175–191. 10.3758/BF03193146 17695343

[B20] Furedy J. J. Posner R. T. Vincent A. (1991). Electrodermal differentiation of deception: perceived accuracy and perceived memorial content manipulations. Int. J. Psychophysiol. 11, 91–97. 10.1016/0167-8760(91)90376-9 1856118

[B21] Gamer M. Gödert H. W. Keth A. Rill H.-G. Vossel G. (2008). Electrodermal and phasic heart rate responses in the Guilty Actions Test: comparing guilty examinees to informed and uninformed innocents. Int. J. Psychophysiol. 69, 61–68. 10.1016/j.ijpsycho.2008.03.001 18433904

[B22] Gamer M. Rill H. G. Vossel G. Gödert H. W. (2006). Psychophysiological and vocal measures in the detection of guilty knowledge. Int. J. Psychophysiol. 60, 76–87. 10.1016/j.ijpsycho.2005.05.006 16005091

[B23] Good A. J. (1967). Letters and Comments. J. Parapsychol. 35, 57–58.

[B24] Gronau N. Ben Shakhar G. Cohen A. (2005). Behavioral and physiological measures in the detection of concealed information. J. Appl. Psychol. 90, 147–158. 10.1037/0021-9010.90.1.147 15641895

[B25] Hartwell J. W. (1978). Contigend negatige variation as an index of precognitive information. Eur. J. Parapsychol. 42, 83–85.

[B26] Honorton C. Ferrari D. C. (1989). Future telling”: a meta-analysis of forced-choice precognition experiments, 1935–1987. J. Parapsychol. 53, 1–308.

[B27] Jennings J. R. Hall S. W. (1980). Recall, recognition, and rate: memory and the heart. Psychophysiology 17, 37–46. 10.1111/j.1469-8986.1980.tb02457.x 7355188

[B28] Kircher J. C. Raskin D. C. (2003). The Computerized Polygraph System II (Software Version 4.01). Salt Lake City, UT: Scientific Assessment Technologies, Inc.

[B29] Levin J. Kennedy J. (1975). The Relationship of Slow Cortial Potentials to Psi Information in Man. Presented at the Southeastern Regional Parapsychological Association, Durham, NC.

[B30] Lykken D. T. (1959). The GSR in the detection of guilt. J. Appl. Psychol. 43, 385–388. 10.1037/h0046060

[B31] Lykken D. T. Venables P. H. (1971). Direct measurement of skin conductance: a proposal for standardization. Psychophysiology 8, 656–672. 10.1111/j.1469-8986.1971.tb00501.x 5116830

[B32] May E. C. Paulinyi T. Vassy Z. (2005). Anomalous anticipatory skin conductance response to acoustic stimuli: experimental results and speculation about a mechanism. J. Altern. Complement. Med. 11, 695–702. 10.1089/acm.2005.11.695 16131294

[B33] McCraty R. Atkinson M. Bradley R. T. (2004). Electrophysiological evidence of intuition: part 2. A system-wide process? J. Altern. Complement. Med. (New York, N.Y.), 10, 325–336. 10.1089/107555304323062310 15165413

[B34] Meijer E. H. Selle N. K. Elber L. Ben-Shakhar G. (2014). Memory detection with the concealed information test: a meta analysis of skin conductance, respiration, heart rate, and P300 data: CIT meta-analysis of SCR, respiration, HR, and P300. Psychophysiology 51, 879–904. 10.1111/psyp.12239 24916920

[B35] Mossbridge J. A. Tressoldi P. Utts J. Ives J. A. Radin D. Jonas W. B. (2014). Predicting the unpredictable: critical analysis and practical implications of predictive anticipatory activity. Front. Hum. Neurosci. 8:146. 10.3389/fnhum.2014.00146 24723870 PMC3971164

[B36] Mossbridge J. Tressoldi P. Utts J. (2012). Predictive physiological anticipation preceding seemingly unpredictable stimuli: a meta-analysis. Front. Psychol. 3:390. 10.3389/fpsyg.2012.00390 23109927 PMC3478568

[B37] Mossbridge J. Tressoldi P. Utts J. Ives J. A. Radin D. Jonas W. B. (2015). We Did See This Coming: Response to, We Should Have Seen This Coming, by D. Sam Schwarzkopf. Available online at: http://arxiv.org/abs/1501.03179

[B38] Radin D. Borges A. (2009). Intuition through time: what does the seer see? Explore J. Sci. Heal. 5, 200–211. 10.1016/j.explore.2009.04.002 19608110

[B39] Radin D. I. (1997). Unconscious perception of future emotions: an experiment in presentiment. J. Sci. Explor. 11, 163–180.

[B40] Radin D. I. (2004). Electrodermal presentiments of future emotions. J. Sci. Explor. 18, 253–273.

[B41] Radin D. I. (2006). Psychophysiological evidence of possible retrocausal effects in humans. Front. Time Retrocausation Exp. Theor. 863, 193–213. 10.1063/1.2388755

[B42] Radin D. I. Vieten C. Michel L. Delorme A. (2011). Electrocortical activity prior to unpredictable stimuli in meditators and nonmeditators. Explore J. Sci. Heal. 7, 286–299. 10.1016/j.explore.2011.06.004 21907152

[B43] Radin D. Lobach E. (2007). Toward understanding the placebo effect: investigating a possible retrocausal factor. J. Altern. Complement. Med. 13, 733–740. 10.1089/acm.2006.6243 17931066

[B44] Ritchie S. J. Wiseman R. French C. C. (2012). Failing the future: three unsuccessful attempts to Replicate Bem's “Retroactive Facilitation of Recall” effect. PLoS ONE 7:e33423 10.1371/journal.pone.0033423 22432019 PMC3303812

[B45] Schönwetter T. Ambach W. Vaitl D. (2011). Does a modified guilty knowledge test reveal anomalous interactions within pairs of participants? J. Parapsychol. 75, 93.

[B46] Schwarzkopf D. S. (2014). We should have seen this coming. Front. Hum. Neurosci. 8:332. 10.3389/fnhum.2014.00332 24904372 PMC4034337

[B47] Sokolov E. N. (1963). Higher nervous functions: the orienting reflex. Annu. Rev. Physiol. 25, 545–580. 10.1146/annurev.ph.25.030163.002553 13977960

[B48] Thomaschke R. Dreisbach G. (2013). Temporal predictability facilitates action, not perception. Psychol. Sci. 24, 1335–1340. 10.1177/0956797612469411 23698617

[B49] Timm H. W. (1982). Analyzing deception from respiration patterns. J. Police Sci. Adm. 10, 47–51.

[B50] Tressoldi P. E. Martinelli M. Semenzato L. Cappato S. (2011). Let your eyes predict: prediction accuracy of pupillary responses to random alerting and neutral sounds. SAGE Open, 1, 1–7. 10.1177/2158244011420451

[B51] Velden M. Wölk C. (1987). Depicting cardiac activity over real time: a proposal for standardization. J. Psychophysiol. 1, 173–175.

[B52] Verschuere B. Ben-Shakhar G. Meijer E. H. (2011). Memory Detection. Cambridge: Cambridge University Press.

[B53] Wackermann J. (2002). On cumulative effects and averaging artefacts in randomised S-R experimental designs, in Presented at the 45th Annual Convention of the Parapsychological Association, Paris.

[B54] Wagenmakers E.-J. Wetzels R. Borsboom D. van der Maas H. L. J. (2011). Why psychologists must change the way they analyze their data: the case of psi: comment on Bem (2011). J. Pers. Soc. Psychol. 100, 426–432. 10.1037/a0022790 21280965

